# Impact of borderline personality disorder traits on the association between age and health-related quality of life: A cohort study in the general population

**DOI:** 10.1192/j.eurpsy.2021.27

**Published:** 2021-04-26

**Authors:** L. Botter, M. ten Have, D. Gerritsen, R. de Graaf, S. D. M. van Dijk, R. H. S. van den Brink, R. C. Oude Voshaar

**Affiliations:** 1 Department of Psychiatry, University of Groningen, University Medical Center Groningen, Groningen, The Netherlands; 2 Atlant, Markenhaven, Center for Specialized Chronic Psychiatric Nursing Home Care, Beekbergen, The Netherlands; 3 Trimbos Institute, Netherlands Institute of Mental Health and Addiction, Utrecht, The Netherlands; 4 Department of Primary and Community Care, Radboud University Medical Center, Radboud Institute for Health Sciences, Nijmegen, The Netherlands

**Keywords:** Aging, borderline personality disorder, older adults, quality of Life

## Abstract

**Background:**

Increasing age as well as borderline personality pathology are associated with a lower level of health-related quality of life (HR-QoL). Our objective was to investigate whether the presence of borderline personality traits modifies the association between age and HR-QoL in the general population.

**Methods:**

Cross-sectional data from 5,303 respondents (aged 21–72 years) of the Netherlands Mental Health Survey and Incidence Study-2 were analyzed. Borderline personality traits were assessed with the International Personality Disorder Examination questionnaire. Mental and physical HR-QoL were measured with the Medical Outcomes Study Short Form Health Survey. Multiple linear regression analysis was used to examine the association of borderline personality traits, age and their interaction on mental as well as physical HR-QoL, adjusted for demographic variables as well as somatic and mental disorders.

**Results:**

A total of 1,520 (28.7%) respondents reported one or more borderline personality traits of which 58 (1.1%) reported five or more indicative of a borderline personality disorder. A higher age was associated with lower physical HR-QoL. This negative association became significantly stronger in the presence of borderline personality traits. The association between increasing age and mental HR-QoL was positive in the absence of borderline personality traits and negative in the presence of borderline personality traits.

**Conclusion:**

Borderline personality traits negatively interfere with the association between age and HR-QoL irrespective of somatic and mental disorders. Attention of clinicians and researchers for subthreshold borderline personality pathology is needed in middle-aged and older persons.

## Introduction

Borderline personality disorder was long regarded as a static and untreatable condition, but the perspective has shifted toward recognition of its changeable nature. Similar to healthy personality characteristics, their pathological counterparts turned out to be subject to development across the life span [1]. Despite apparent changes in prevalence and symptomology over time, disease burden of borderline personality disorder is tremendous and remains impactful throughout the life course. For instance, functional impairments and comorbid somatic and mental disorders are commonly found in borderline personality disorder [2, 3] and are pervasive in both younger and older patients [4–7]. Even subthreshold personality disorders, such as borderline personality traits, are associated with these negative consequences [8]. Quality of life (QoL) is increasingly viewed as a main indicator of disease burden and an important aspect of treatment outcome [9,10]. In order to further understand the variform impact of borderline personality traits across the life span, we focus our study on the relation between borderline personality traits, age, and QoL.

Healthy personality traits may explain up to 45% variance in mental and up to 39% of variance in physical health-related (HR)-QoL [11]. However, studies on the relationship between pathological personality traits and QoL in the general population are quite scarce. Typically, samples consist of psychiatric patients and moreover lack inclusion of adults older than 65 years [7]. A systematic review containing predominantly studies on patient populations suggests that borderline personality disorder is associated with a lower QoL in young and middle-aged adults [9]. In later life, borderline personality disorder is associated with several age-related conditions like somatic comorbidity [12], higher risk for serious life events [13] and more functional impairment than younger adults with borderline personality disorder [7]. This underscores that when studying the impact of borderline personality disorder across the life span, it is vital to include older persons in the sample, as well as persons with subthreshold levels of borderline personality disorders (i.e., borderline personality traits).

Borderline personality traits appear to amplify the overall impact of an increasing age on QoL. Nonetheless, empirical data on the bivariate association between borderline personality traits and QoL in older adults are particularly scarce [12,13]. For instance, among outpatients with borderline personality disorders, QoL was significantly lower among patients aged 46 years and older compared to younger patients [14]. Among older patients with depression, cluster B personality traits (including borderline traits) were found to be negatively associated with QoL [15]. This effect remained when the depression was remitted at 1-year follow-up, albeit the effect size was small and not adjusted for potential confounders such as physical and mental health indices. Other studies in older adults demonstrate direct associations between personality disorders and QoL, but these studies did not intentionally include patients with borderline personality disorder [16,17]. It can be concluded that empirical findings are thus far insufficient to make distinct inferences about the impact of borderline personality traits on the association between age and HR-QoL in the general population.

The prevalence of a personality disorder according to psychiatric diagnostic criteria is thought to become lower with increasing age [18]. Nonetheless, the prevalence of any personality disorder is still estimated at 8.1–11.4% among persons aged 65 years and over [19,20]. Well-substantiated prevalence rates for borderline personality disorder in older adults are scarce. Similar to young adults, cluster B personality disorders (including borderline personality disorder) are less common among older people than personality disorders in cluster A and C [21,22]. Data from the second wave of the comprehensive NESARC study also demonstrate a decline in all personality disorders in older adults, including borderline personality disorder [20]. The AUDADIS-IV was used as the assessment tool to examine all psychiatric disorders whereas in the first NESARC wave the International Personality Disorder Examination (IPDE) was used. Despite the overall decline across age groups, the found prevalence rate of borderline personality disorder for adults older than 55 (3.2%) is remarkably higher than that of adults in general (commonly estimated around 1% [2,8]). However, despite the impressive sample, this remains the finding of a single study. Multiple explanations have been put forward for the decline in borderline personality disorders in older adults, such as high morbidity and mortality due to risk behavior, suicide and somatic comorbidity [23,24], and a decrease in impulsive and aggressive behavior when comes to age. For personality disorders in general, it has been demonstrated that 29% of the diagnostic criteria are not age neutral, which could lead to under detection of personality disorders in later life [25]. If indeed borderline personality disorder in later life is harder to detect but still impactful [18], it would be valuable to further comprehend the consequences of possessing borderline personality traits throughout a person’s life. This is especially relevant considering a meta-analysis showing that in depressed patients, a comorbid personality disorder doubles the odds of a poor outcome for depression compared to those with no personality disorder [26], while psychotherapy for personality disorders in later life appears promising [27].

### Aims of the study

The aim of this study is to investigate whether the presence of borderline personality traits affect the association between age and HR-QoL in the general population. We will specifically distinguish between physical and mental HR-QoL [28]. We hypothesize that physical HR-QoL may be more affected by an increasing age (due to the inevitable increase of the somatic disease burden) than mental HR-QoL (as mental resilience and coping may increase with age [29] and prevalence rates of affective disorders decrease with age [28,30]). Since borderline personality traits interfere with coping abilities and mental health, we hypothesize that negative effects of increasing age on QoL will become stronger in the presence of borderline personality traits.

## Methods

### Sample

The Netherlands Mental Health Survey and Incidence Study-2 (NEMESIS-2) is a representative epidemiological cohort study of the general Dutch population aged 18–65 at the first wave (*N* = 6,646, response rate 65.1%). One participant per household was randomly selected and interviewed at home. The first wave (T_0_) was completed during the period of November 2007 to July 2009. The entire T_0_ group was contacted for follow-up 3 years later and 5,303 respondents (response rate 80.4%, excluding deceased persons) were interviewed again from November 2010 to June 2012 [31]. Data on borderline personality traits were collected at this second wave (T_1_). Attrition at T_1_ was not meaningfully associated with all main categories and individual 12-month mental disorders at T_0_ after controlling for sociodemographic characteristics [31].

The NEMESIS-2 study protocol was approved by a medical ethics committee and all participants provided written informed consent at all waves. For a comprehensive description of the NEMESIS-2 study design, we refer to De Graaf et al. [32].

### Measures

#### Borderline personality traits

The eight questions of the IPDE corresponding with DSM-IV criteria for a borderline personality disorder were used to measure borderline traits [33,34]. The IPDE does not measure one BPD-criterion, namely recurrent suicidal behavior, gestures or threats, or self-mutilating behavior. The IPDE questions are part of the Composite International Diagnostic Interview (CIDI) version 3.0, a structured lay-administered interview [35]. These eight IPDE questions are answered in a true-false format, with the added number of true answers resulting in a total score (range 0–8). The internal consistency was low (*α* = 0.53), but expectedly so because a single IPDE item is designed to measure just one borderline trait. An IPDE score of 5 and higher can be considered as an indication of a BPD, because of the correspondence with the minimum level of required DSM symptoms (5 out of 9) to meet DSM-IV criterion A for a borderline personality disorder diagnosis. The cut-off of 5 out of 8 IPDE questions has repeatedly been demonstrated to be a valid method of assessing the presence of a borderline personality disorder [2,31,34,36].

#### Primary outcome

HR-QoL was assessed with the Medical Outcomes Study Short Form Health Survey (MOS SF-36) [37]. The MOS SF-36 is commonly used in research to measure HR-QoL and has been validated in the general population [38]. This self-report questionnaire consists of 36 items which ask how respondents felt over the last 4 weeks using different Likert scales (2-, 3-, 5- and 6-point Likert scales). The 36 items comprise in total 8 health domains or subscales, which culminate in a well-validated physical and mental component summary score [39].

#### Covariates

All covariates were selected on their putative association with both borderline personality traits and HR-QoL. As demographic characteristics, we included sex, age, and level of education [8]. Next, mood disorder (major depression, dysthymia, or bipolar disorder), anxiety disorder (panic disorder, agoraphobia, social phobia, specific phobia, or generalized anxiety disorder), and substance use disorder (alcohol/drug abuse or dependence) in the last 12 months were considered as three separate potential confounders. The presence of these disorders was assessed using the CIDI 3.0. The CIDI 3.0 is a fully structured psychiatric interview and a psychometrically sound instrument to assess common mental DSM-IV diagnoses in the general population [40]. Finally, the presence of somatic disorders (yes/no), based on self-reported presence of a chronic somatic disease (≥1 of 17 chronic physical disorders treated or monitored by a medical doctor in the past 12 months, assessed with a standard checklist, was considered a potential confounder).

### Statistical analyses

Since the number of borderline personality traits was not normally distributed, neither after log-transformation, nor the number of respondents with five or more traits was low (*n* = 58), descriptive statistics are presented, stratified for persons with no, one, or two or more borderline personality traits. Differences between these three groups on the covariates and HR-QoL were tested with either a *χ*^2^ test (for categorical variables) or one-way ANOVA (for continuous variables).

Next, we examined the association between all covariates as well as borderline personality traits (entered simultaneously as independent variables) with HR-QoL (dependent variable) by multiple linear regression analyses. Separate analyses were conducted for mental and physical HR-QoL.

Next, we examined whether the presence of borderline personality traits interacted with age in explaining variance in HR-QoL. In case of significant interaction (*p* < 0.05), results were stratified according to the presence of no, one, and two or more borderline personality traits. A sensitivity analyses was performed by testing the association between age and HR-QoL, stratified by presence of a borderline personality disorder (≥5 traits) or not. To facilitate clinical interpretation of results, we also explored the interaction between each borderline personality trait and age separately on explaining either physical or mental HR-QoL by multiple linear regression.

Analyses were based on unweighted data as we are primarily interested in associations between characteristics. Data were analyzed using SPSS version 26 [41]. *p*-values of <0.05 are considered statistically significant.

## Results

### Sample characteristics

In the total sample (age range 21–72), 71.3% reported having no borderline personality traits, while 24.1% had 1–2 traits, 3.5% had 3–4 traits, and 1.1% had ≥5 traits. Table 1 presents the baseline characteristics stratified by the numbers of borderline personality traits, that is none (*n* = 3,783, 71.3%), one trait (*n* = 936, 17.7%), or two or more traits (*n* = 584, 11.0%). The presence of borderline personality traits was significantly related to younger age, female sex, lower education level, higher somatic comorbidity, having a mood disorder, anxiety disorder, substance use disorder, and lower mental and physical HR-QoL (see Table 1).

**Table 1.** Baseline characteristics of participants (*n* = 5,303), stratified by the number of borderline personality traits.

	No borderline traits	One borderline trait	Two or more borderline traits	
Characteristics	(*n* = 3,783)	(*n* = 936)	(*n* = 584)	Statistics^a^
Mean (SD) age (years)	48.1 (12.4)	47.0 (12.3)	45.7 (12.2)	*F* = 11.2, df = 2, *p* < 0.001
Female sex, *n* (%)^a^	2,145 (56.7%)	440 (47.0%)	338 (57.9%)	*χ*^2^ = 30.5, df = 2, *p* < 0.001
Level of education				
Lower education, *n* (%)	1,075 (28.4%)	320 (34.0%)	219 (37.5%)	
Middle education, *n* (%)	1,223 (32.3%)	296 (31.6%)	209 (35.8%)	*χ*^2^ = 45.1, df = 2, *p* < 0.001
Higher education, *n* (%)	1,485 (39.3%)	320 (34.2%)	156 (26.7%)	
Somatic comorbidity (any), *n* (%)^a^	1,520 (40.2%)	417 (44.6%)	303 (51.9%)	*χ*^2^ = 30.9, df = 2, *p* < 0.001
Psychopathology (current diagnoses)				
Mood disorder (any), *n* (%)^a^	71 (1.9%)	62 (6.6%)	137 (23.5%)	*χ*^2^ = 493.2, df = 2, *p* < 0.001
Anxiety disorder (any), *n* (%)^a^	126 (3.3%)	60 (6.4%)	129 (22.1%)	*χ*^2^ = 319.1, df = 2, *p* < 0.001
Substance use disorder (any), *n* (%)	64 (1.7%)	43 (4.6%)	49 (8.4%)	*χ*^2^ = 90.4, df = 2, *p* < 0.001
Quality of life (QoL)				
Mean (SD) mental health related QoL^a^	85.5 (11.0)	80.3 (15.1)	67.1 (21.8)	*F* = 499.7, df = 2, *p* < 0.001
Mean (SD) physical health related QoL^a^	84.4 (17.1)	80.3 (19.4)	71.9 (23.3)	*F* = 125.3, df = 2, *p* < 0.001

Abbreviation: SD, standard deviation.

a One-way ANOVA or *χ*^2^-test.

### Association of borderline personality traits with HR-QoL

Multiple linear regression analyses showed that, without consideration of interaction terms, increasing age was related to lower physical HR-QoL but not to mental HR-QoL (see Table 2). Furthermore, an increasing number of borderline personality traits was significantly associated with lower mental HR-QoL and physical HR-QoL. The associations between borderline personality traits and HR-QoL were confirmed by a sensitivity analysis on the presence of BPD (5 or more traits) for both mental HR-QoL (*B* = −19.00 [standard error, SE = 1.75], *β* = −0.14, *p* < 0.001) and physical HR-QoL (*B* = −14.95 [SE = 2.34], *β* = −0.08, *p* < 0.001).

**Table 2.** Multiple linear regression analyses on health-related quality of life.

	Mental health related QoL			Physical health related QoL		
Variables	*B* (SE)	*β*	*p*	*B* (SE)	*β*	*P*
Age	0.02 (0.02)	0.02	.179	−0.07 (0.02)	−0.04	0.001
Female sex	−2.57 (0.35)	−0.08	<.001	−2.72 (0.46)	−0.07	<0.001
Education (lower is reference):						
Middle level	0.36 (0.44)	0.01	.827	3.30 (0.58)	0.08	<0.001
Higher level	−0.52 (0.43)	−0.02	.219	3.51 (0.56)	0.09	<0.001
Somatic comorbidity	−3.59 (0.36)	−0.12	<.001	−13.25 (0.48)	−0.35	<0.001
Psychopathology (past year)						
Depressive disorder	−12.13 (0.84)	−0.18	<.001	−8.20 (1.12)	−0.10	<0.001
Anxiety disorder	−9.60 (0.76)	−0.16	<.001	−5.84 (1.01)	−0.07	<0.001
Substance use disorder	−3.58 (1.02)	−0.04	<.001	−2.65 (1.36)	−0.02	0.050
Borderline personality traits (none = ref)						
One trait	−4.25 (0.46)	−0.11	<.001	−2.99 (0.61)	−0.06	<0.001
Two or more traits	−12.91 (0.60)	−0.28	<.001	−7.35 (0.79)	−0.12	<0.001

Abbreviations: QoL, quality of life; SE, standard error.

### Interaction with age

Multiple linear regression analyses yielded a statistically significant interaction between borderline personality traits and age on the association with mental HR-QoL (one trait: *p* = 0.044; two or more traits: *p* < 0.001) as well as physical HR-QoL (one trait: *p* = 0.094; two or more traits: *p* < 0.022) in the fully adjusted models. Therefore, Table 3 presents the association between age and HR-QoL stratified by the number of borderline personality traits.

**Table 3.** Association between age and HR-QoL by multiple linear regression,^a^ stratified by the number of borderline personality traits.

	Mental HR-QoL			Physical HR-QoL		
Variables	*B* (SE)	*β*	*p*	*B* (SE)	*β*	*p*
No borderline personality traits						
Age	0.06 (0.02)	0.07	<0.001	−0.04 (0.02)	−0.03	0.057
One borderline personality trait						
Age	−0.06 (0.04)	−0.05	0.168	−0.14 (0.05)	−0.09	0.006
Two or more borderline personality traits						
Age	−0.10 (0.07)	−0.06	0.148	−0.13 (0.08)	−0.07	0.082

Abbreviation: HR-QoL, health-related quality of life.

a Adjusted for sex, level of education, somatic comorbidity, and psychopathology (presence of a mood disorder, presence of an anxiety disorder and presence of a substance use disorder).

The stratified analyses showed that among persons with no borderline personality traits, mental HR-QoL is better in older individuals compared to younger individuals. In contrast, among persons with one or two or more borderline personality traits, higher age was associated with a lower mental HR-QoL, albeit these effects were not significant. With respect to physical HR-QoL, irrespective of the number of borderline personality traits, an increasing age was associated with a worse HR-QoL. The impact of age, however, was lowest in the subgroup of persons without any borderline personality trait (see Table 3).

A sensitivity analysis of age by the presence of a borderline personality disorder (5 or more traits present, *n* = 58), also yielded a significant interaction of borderline by age with mental-HR-QoL (*p* = 0.003) as well as with physical HR-QoL (*p* = 0.033). Stratified analyses among persons with a borderline personality disorder showed a strong negative association between age and HR-QoL, although not statistically significant (mental HR-QoL: *B* = −0.29 [SE = 0.31], *β* = −0.13, *p* = 0.348; physical HR-QoL: *B* = −0.26 [SE = 0.31], *β*= −0.10, *p* = 0.417), in contrast to persons without BPD (mental HR-QoL: *B* = 0.04 [SE = 0.02], *β* = 0.04, *p* = 0.004; physical HR-QoL: *B* = −0.05 (SE = 0.02), *β* = −0.04, *p* = 0.007).

Post-hoc, we tested whether the association between the eight specific borderline personality traits interacted with age in explaining variance in either mental or physical HR-QoL. We found that four traits significantly interacted with age in explaining mental HR-QoL, that is chronic feelings of emptiness (*p* = 0.002), affective instability (*p* = 0.006), inappropriate, intense anger or difficulty controlling anger (*p* < 0.001) and transient, stress-related paranoid ideation or dissociative symptoms (*p* < 0.001). Furthermore, we also found four traits that significantly interacted with age in explaining physical HR-QoL, that is chronic feelings of emptiness (*p* < 0.001), affective instability (*p* = 0.031), impulsivity (*p* = 0.045), and transient, stress-related paranoid ideation or dissociative symptoms (*p* < 0.001). Table 4 presents the associations of age with either mental or physical HR-QoL stratified for the presence of the particular traits, for those traits which showed a significant interaction with age. Regarding mental HR-QoL, in persons with these specific personality traits increasing age is not associated with a better mental HR-QoL anymore. Regarding physical HR-QoL, in persons with these specific traits the association between increasing age and a lower physical HR-QoL becomes significantly stronger.

**Table 4.** Association between age and HR-QoL by multiple linear regression,^a^ stratified by the presence of a specific borderline personality trait.^b^

	Mental HR-QoL			Physical HR-QoL		
Variables	*B* (SE)	*β*	*p*	*B* (SE)	*β*	*p*
Chronic feelings of emptiness						
No: Age	0.06 (0.01)	0.06	<0.001	−0.04 (0.02)	−0.03	0.028
Yes: Age	−0.07 (0.11)	−0.04	0.534	−0.19 (0.12)	−0.09	0.102
Affective instability						
No: Age	0.04 (0.02)	0.04	0.009	−0.05 (0.02)	−0.03	0.014
Yes: Age	−0.07 (0.09)	−0.04	0.447	−0.16 (0.10)	−0.09	0.111
Impulsivity						
No: Age	n.a.			−0.05 (0.02)	−0.04	0.009
Yes: Age	n.a.			−0.14 (0.12)	−0.07	0.249
Inappropriate, intense anger						
No: Age	0.04 (0.02)	0.04	0.006	n.a.		
Yes: Age	−0.10 (0.07)	−0.06	0.151	n.a.		
Transient paranoia or dissociation						
No: Age	0.05 (0.02)	0.04	0.002	−0.05 (0.02)	−0.03	0.015
Yes: Age	0.21 (0.10)	−0.12	0.028	−0.28 (0.10)	−0.15	0.006

Abbreviations: HR-QoL, health-related quality of life; n.a., not applicable; SE, standard error.

a Adjusted for sex, level of education, somatic comorbidity, and psychopathology (presence of a mood disorder, presence of an anxiety disorder and presence of a substance use disorder).

b Only criteria with a significant interaction with age are presented.

## Discussion

To the best of our knowledge, this is the first study presenting empirical findings on the impact of borderline personality traits on the relationship between age and HR-QoL in the general population including older persons. Previous studies on personality disorders in different life stages and QoL were confined to psychiatric samples [14], the physical component of HR-QoL [17], or typically lacked respondents with BPD or established borderline personality traits [15,17]. Furthermore, adults older than 65 years were entirely absent in most samples [7]. The present study demonstrates that presence of borderline personality traits modifies the association between age and both aspects of HR-QoL in line with our hypotheses. Older individuals possess a significantly better mental HR-QoL than younger adults, however, in the presence of one or more borderline personality traits, an increasing age was not associated with better mental HR-QoL anymore. This remained the case after controlling for mental disorders that frequently co-occur with borderline personality traits [8]. Furthermore, physical HR-QoL turned out to become lower with increasing age, but even more so in the presence of borderline personality traits. The association between age and HR-QoL was strongest among persons meeting the criteria for a borderline personality disorder, although not statistically significant due to the low number of respondents meeting the criteria for a borderline personality disorder. Nonetheless, even the presence of one borderline personality trait already resulted in differential associations between age and HR-QoL. This suggests that growing older with any number of borderline personality traits is associated with a higher level of mental and physical health issues, which has a negative impact on the QoL. Our results are in line with a previous study reporting that among patients with borderline personality disorder age was associated with a lower level of mental and physical HR-QoL [14].

We found that among persons without borderline personality traits an increasing age is associated with a higher mental HR-QoL. This contrasts with studies in the literature that describe a stable HR-QoL across different life stages, but these studies did not stratify by the presence of borderline personality traits [42]. Moreover, most previous studies may have been confounded by comorbid mental and somatic disorders. We adjusted for these comorbidities to estimate the impact of age itself on mental HR-QoL scores. The finding that older adults do not experience a decline in mental HR-QoL may be explained in the first place by older adults possessing better (passive) emotional coping skills that protect them when experiencing serious/negative life events such as bereavement or loss of a professional life [43]. As a deficiency in emotional coping skills is one of the core symptoms of borderline personality disorder, it is conceivable that older adults with borderline personality traits do not profit from the common development of emotional skills associated with aging [8]. Accordingly, events such as widowhood and divorce are indeed associated with a lower QoL in older adults with a personality disorder [16]. Another explanation is that borderline personality traits in themselves lead to more negative interpersonal life events which subsequently affect QoL [13]. Lastly, interaction between age and number of borderline personality traits on mental health related QoL might be confounded by differences in severity of particular traits between younger and older persons. One may imagine that milder traits may improve over time, while more severe traits may persist lifelong and have a stronger impact on QoL. Nonetheless, to explore this hypothesis the individual borderline personality traits should have been assessed dimensionally (for which valid assessment instruments are lacking).

Lower physical HR-QoL in older adults has also been associated with having a dependent, obsessive–compulsive or paranoid personality disorder [17]. In the present study, we demonstrated that even subthreshold levels of borderline personality traits are related to lower physical HR-QoL when controlled for somatic comorbidity. Several explanations can be put forward. First, the presence of borderline personality traits may negatively interfere with coping with chronic somatic diseases. Secondly, personality disorders in general are related to a broad range of physical health problems, such as sleeping problems and physical pain, which do not necessarily translate into somatic disorders [44]. Moreover, borderline-related behavior such as auto-mutilation, suicide attempts, physical harm from impulsive decision making, and substance abuse may further contribute to physical disability, bad health behavior or worsen the course of already present chronic somatic diseases [45].

These results can have several practical implications, pertaining to various stages of life. Firstly, the lifelong harmful physical and mental effects emphasize that possession of borderline personality traits needs to be viewed as a serious risk factor for a person’s wellbeing throughout life. Borderline personality traits should be targeted by health policies and health care professionals from early on. Timely recognition of borderline personality traits and providing of the appropriate treatment may prevent the occurrence of comorbid physical and mental health problems that would otherwise accumulate with aging. Moreover, attention for all aspects of a healthy life (e.g., life style, physical health, and self-management) is justified, as has been advocated by others [45–47]. Secondly, as pertaining to later life stages, it would be erroneous to assume that absence of clinically established borderline personality disorder in older adults equals the absence of borderline-related health problems. It appears that while some behavioral borderline-related symptoms may diminish across the life span, underlying pathological personality traits remain stable. This concept is described as heterotypical continuity and is supported by several literature reviews [48–50]. It is conceivable that this would easily lead to under detection of borderline personality disorders in older adults, while missing clinically relevant personality pathology [22]. This is even more relevant knowing the impact of subthreshold levels of borderline traits. As appears from our results of the specific borderline criteria, especially traits in the affective-internalizing domain such as chronic feelings of emptiness or affective instability may be considered as indicators of underlying pathology that is related to a lower HR-QoL with an increasing age (see Table 4). Studies that discuss the idea of heterotypical continuity also identify affective symptoms as the temporally stable traits in older BPD patients, as opposed to externalizing traits [18]. Our finding that low levels of borderline traits negatively affect older adults might also have implications for treatment. If affective symptoms reported by an older patient are indeed persistent and related to borderline personality traits, long-term insightful psychotherapy might be preferred over short-term symptom-based therapy. Schema therapy for example appears to be a good option, as the evidence of effectivity in older adults is taking shape [27]. In order to investigate the possibility that older adults with borderline personality traits can attain better levels of QoL by means of psychotherapy, more empirical research is needed.

Several limitations apply to the interpretation of the results as described above. Firstly, the described associations cannot be causally interpreted due to the cross-sectional nature of this study. Secondly, this study did not include socioeconomic factors that might serve as determinants of QoL in later life and might either explain or confound the association with borderline personality traits [51]. Since socioeconomic status is generally lower in the older population, our results may be even conservative estimates. Thirdly, while the IPDE can be considered a good psychometric instrument for general research purposes, the use of a self-report instrument might have led to underreporting of borderline personality traits [52,53]. This has probably attenuated our findings. The addition of semi-structured interviews allowing interpretation of answers by well-trained clinicians would increase diagnostic accuracy of borderline personality traits.

### Final conclusion

Borderline personality traits, even when the criteria for a full borderline personality disorder are not met, modify the association between age and HR-QoL. The presence of borderline personality traits precludes the increase of mental HR-QoL with age and results in a stronger decline in physical HR-QoL with age. Therefore, borderline personality traits should be treated as markers of serious health problems across the life span. Also, more attention is warranted from clinicians and researchers for subthreshold personality problems in middle-aged and older persons, in order to increase understanding of their impact on HR-QoL and improve diagnostics and treatment options in clinical practice. So that, hopefully, older patients also, instead of suffering ever more, can reap the benefits of aging.

## Abbreviations

BPD: borderline personality disorder
HR-QoL: health-related quality of life
CIDI: Composite International Diagnostic Interview
IPDE: International Personality Disorder Examination
MOS SF-36: Medical Outcomes Study Short Form Health Survey 36
NEMESIS-2: Netherlands Mental Health Survey and Incidence Study-2
QoL: quality of life

## Data Availability

The datasets generated during and/or analyzed during the current study are available from the corresponding author on reasonable request.
